# Supplementary material for: Cancer patient survival can be parametrized to improve trial precision and reveal time-dependent therapeutic effects
Source: Nat Commun. 2022 Feb 15;13:873. doi: 10.1038/s41467-022-28410-9 (PMC8847344; doi:10.1038/s41467-022-28410-9)
Supplement: Supplementary file 5 — Supplementary Dataset 2 [file 41467_2022_28410_MOESM5_ESM.zip › Supplementary Data File 2/Weibull all fits survival plots- Fig 1, 2, S1, S2/MA31_2_ltax_l.pdf]

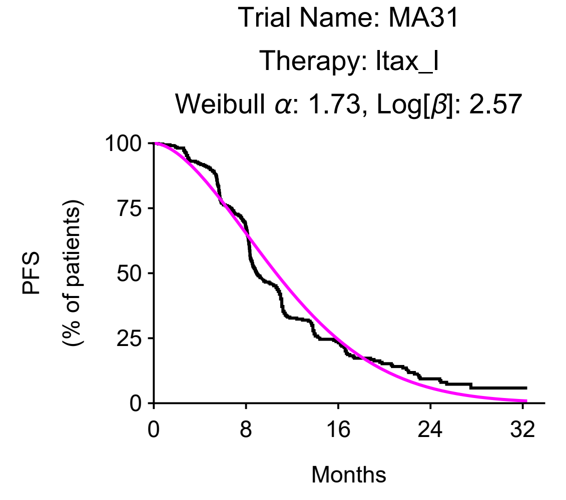
Trial Name: MA31
Therapy: Itax_l
Weibull α: 1.73, Log[β]: 2.57
100
75
50
25
0
PFS
(% of patients)
0
8
16
24
32
Months
